# Richard P. Michael, MD, PhD, DSc, FRCPsych Formerly Reader in Behavioural Physiology and Consultant Psychiatrist at the Bethlem Royal and Maudsley Hospital (1963–1972), Professor of Psychiatry and Anatomy, Emory University School of Medicine, Atlanta, USA

**DOI:** 10.1192/pb.bp.114.049593

**Published:** 2014-12

**Authors:** Gerald Russell

**Figure:**
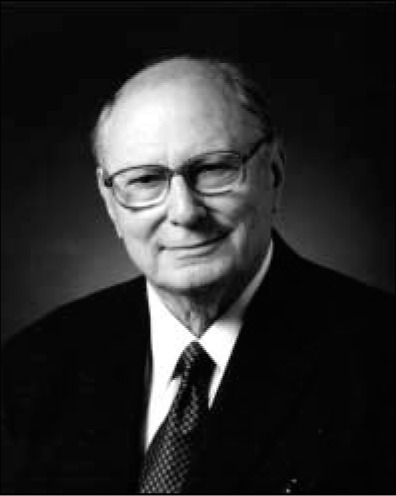


Richard Michael, who died recently at the age of 89, gained a reputation for his meticulous and ground-breaking studies of the neuroendocrine mechanisms underlying sexual behaviour, a subject highly relevant to clinical and social psychiatry. These studies involved the observation of sexual behaviour in female cats and correlating it with stages of the oestrous cycle.

His most productive research was done at an early stage at the Institute of Psychiatry, London. He began his appointment there in 1959, formally as a member of the Department of Psychiatry under Professor Aubrey Lewis but with a close attachment to the Department of Physiology, led by Professor Geoffrey Harris, his principal research mentor. He began with a key observation that the ovariectomised cat never showed receptive sexual behaviour towards the male. This could be re-established with the administration of oestrogens. He was able to demonstrate by carrying out experiments on spayed cats that their mating behaviour could be restored with the systemic administration of oestrogen. The more important aim of this work was to identify the precise location of oestrogen sensitivity in the brain. The hypothesis was that some regions would be highly responsive to tiny concentrated oestrogen implants on the tip of a needle inserted stereotaxically in selected areas. If implanted in a critical area of the brain the oestrogen pellet would restore the stereotyped mating behaviour of the female cat in spite of the animal retaining an anoestrous genital tract. This would demonstrate that the effect of the oestrogen was localised in the brain and was not due to a generalised endocrine effect.

Using meticulous experimental designs, Richard was able to demonstrate that the critical areas lay in the cat’s hypothalamus. This formed part of a ‘mating system’, localised to some degree but stretching from the anterior preoptic region to the post-mammillary bodies. He cautiously concluded in an article in 1962 that this area of the hypothalamus contained oestrogen-sensitive receptors. At this time the notion of an oestrogen receptor in the brain was entirely novel.

Conscious of the importance of species-confined behaviour, Richard then decided to search for equivalent neuroendocrine mechanisms in primates and spent the next 30 years on this endeavour. The rhesus monkeys presented more complex methodological problems. The variability of the monkey’s skull made it difficult to perform stereotaxis accurately. It turned out that the pattern of receptive sexual behaviour of rhesus females was nowhere near as stereotyped as that of female cats. Primates showed large individual differences in behaviour, with marked partner preferences. He concluded, with some reluctance, to leave the development of implant studies in primates to others.

Richard’s research then changed direction so that he conducted mainly behavioural studies. These required the construction of special laboratories situated in the grounds of Bethlem Royal Hospital. He obtained the funding and was directly concerned with the design and building work for the project. A later development was the provision of an outside enclosure for anubis baboons, the only outside experimental primate enclosure in London. He trained research workers in the skills of behavioural observation. A great deal of this work was done in collaboration with Professor Doris Zumpe and they co-authored two books.

It was with some regret that in 1972 Richard left Bethlem for a new appointment as Director of Biological Psychiatry Research Laboratories at the Georgia Mental Health Institute, Atlanta. He found this necessary because of the need for increased funding for this type of research, the animals having become very expensive and requiring first-class facilities for their care and observations. In Atlanta he widened his studies into other aspects of sexual behaviour in primates, such as grooming and the role of olfactory communication and social factors in sexual and aggressive behaviour.

It is not clear what led Richard to develop his keen interest in animal behaviour. He was born in London on 9 June 1924 where he was brought up, attending the Haberdashers’ Aske’s Boys’ School. He was grateful to his teachers for recognising his potential and turning him from what he described as an idle pupil into a high achiever. At an early stage of his medical career, Richard went to the Maudsley where he combined his psychiatric training with clinical and animal research supported by a series of research fellowships. From 1956–1959 he held an MRC Fellowship in clinical research with attachments to the Departments of Physiology at the Royal Free Hospital School of Medicine and the Institute of Psychiatry. In 1958 he obtained a Rockefeller travelling fellowship in medicine, which he held at the National Institute of Mental Health, Bethesda, Maryland, USA. Unusually for a psychiatrist whose main research focus involved physiological studies, throughout his career he maintained a small psychoanalytic practice.

Richard was a remarkable personality with a mischievous sense of humour. Even after years spent in the USA during his later career, he never lost his clear-cut English accent and precise diction. He was hospitable and an excellent host. One of his quirks was to quiz waiters and waitresses in restaurants about their country of origin. This could be embarrassing for his friends but the recipients seemed to enjoy it. His regret at leaving England was mitigated after his retirement by spending half the year in Atlanta and the remainder in his attractive home and garden in Alfriston, East Sussex.

He had a long and happy marriage to Anne Huntington whom he met when they both worked at the Royal Free Hospital. Richard died peacefully at home in Atlanta on 5 January 2014. He is survived by Anne, three sons, one daughter and nine grandchildren.

